# A Composite Membrane System with Gold Nanoparticles, Hydroxyapatite, and Fullerenol for Dual Interaction for Biomedical Purposes

**DOI:** 10.3390/membranes11080565

**Published:** 2021-07-27

**Authors:** Anna Grzeczkowicz, Monika Drabik, Agata Lipko, Paweł Bącal, Angelika Kwiatkowska, Beata Kazimierczak, Ludomira H. Granicka

**Affiliations:** 1 Nalecz Institute of Biocybernetics and Biomedical Engineering, Polish Academy of Sciences, Trojdena 4 St., 02-109 Warsaw, Poland; agrzeczkowicz@ibib.waw.pl (A.G.); mdrabik@ibib.waw.pl (M.D.); alipko@ibib.waw.pl (A.L.); pbacal@ibib.waw.pl (P.B.); ankwiatkowska@op.pl (A.K.); bkazimierczak@ibib.waw.pl (B.K.); 2 Institute of Paleobiology, Polish Academy of Sciences, Twarda 51/55 St., 02-109 Warsaw, Poland

**Keywords:** polyelectrolyte multilayered membranes, osteoblasts, fibroblasts

## Abstract

*Background:* Wound dressing plays a vital role in post-operative aftercare. There is the necessity to develop dressings for application on the border of soft and hard tissue. This study aimed to develop multifunctional polyelectrolyte layers enhanced by hydroxyapatite nanoparticles, gold nanoparticles (AuNPs), and/or fullerenol nanocomposites to achieve a wound dressing that could be applied on the bone-skin interface. *Methods*: Constructed shells were examined using TEM, STEM, and EDX techniques. The human osteoblasts or fibroblasts were immobilized within the shells. The systems morphology was assessed using SEM. The functioning of cells was determined by flow cytomery. Moreover, the internalization of AuNPs was assessed. *Results*: Involvement of fullerenol and/or hydroxyapatite nanoparticles influenced the immobilized cell systems morphology. Membranes with fullerenol and hydroxyapatite nanoparticles were observed to block the internalization of AuNPs by immobilized hFOB cells. *Conclusions*: The designed bilayer membranes incorporating fullerenol, and bacteriostatic elements, prevented the internalization of AuNPs by hFOB cells and ensured the proper counts and morphology of eukaryotic cells. The developed material can be recommended for dressings at the bone-skin interface.

## 1. Introduction

Appropriate post-operative treatment of soft tissue defects with exposed bone surfaces in the face and head region reduces potential complications facilitating further reconstruction. In some tumors like malignant melanomas or basaliomas, the resection is usually extensive, resulting in defects unreachable to primary wound closure [1]. Such defects are challenging to maintain in good condition until the final plastic surgery, especially in the group of elderly patients and among people with comorbidities [2]. Obesity, diabetes, and smoking can increase the risk of infections and prolong the healing time [3], while poorly healed wounds lead to unsatisfying results of further treatment [1].

Facial reconstruction is applied to replace and restore hard and soft tissues, allowing the patient to overcome the trauma and return and re-adapt to society. Moreover, it is often required to recreate the original function of lost organs and structures [4]. Among the most common causes, the restoration of normal deglutition with pharyngeal and tongue components, providing the patency and airway support after a tracheotomy, and the reconstruction of the general movement of the neck and head region might be enumerated [5]. Sometimes the reconstruction must be delayed to months after the original resection. Many factors are forcing this approach, like the risk of seeding cancer cells in newly cut tissues, the possibility of graft infection, or covering of the primary site impeding the detection of a recurrence [6]. Whatever the reason, the medical team has to take care of the wound between the initial and final operations to maintain wounds in good condition [1]. It should be noted that improper treatment causing soft tissue contraction and significant fibrosis, affecting the subsequent restoration and worsening the possibility of the reconstruction.

Wound dressing plays a crucial role in post-operative aftercare. Dressings can effectively support wound management by preventing haematomas, surgical-site infection (SSIs), and wound dehiscence [7]. Generally, modern wound dressings should be adherent to the skin, show antimicrobial properties, be capable of removing redundant exudate, and provide a moist environment promoting healing [8]. Nonetheless, the type of the applied dressing must be chosen depending on the specific case based on the location [9], depth, size of the tissue defect, and exudate degree [7,10]. In addition, each dressing applied for defects on the border of soft and hard tissue used in the surgery therapy must be considered individually to define the characteristics needed to provide the appropriate material behaviour on the bone-skin interface.

Wound treatment immediately after the surgery is critical for the whole healing process and determines the effectiveness of the therapy. Unfortunately, most wounds with exposed bones are hard to heal. It was observed that even if the proper moisture balance is preserved thanks to the application of the modern dressing based on hydrogels and hydrocolloids, the granulation tissue formation for such defects is slow or does not occur at all [11]. Therefore, wounds of this kind cannot be healed by secondary intention and required advanced therapies like the application of additional surgical treatment [12], usage of bioengineered skin substitutes [13,14], or the inclusion of negative pressure wound therapy (NPWT) combined with skin grafts [15,16,17]. However, the high cost of the techniques mentioned above and the necessity of general anaesthesia during the operation make them inaccessible for medically compromised and elderly part of the population. Therefore, alternative treatment methods are constantly being sought. One of such approaches assesses the usage of wound therapeutics, usually in the form of foam, the example of which can be Swiss drug 1 PRIMARY WOUND DRESSING^®^ [ONE] (produced by Kerecis AG, Adliswil, Switzerland, formerly Phytoceuticals AG,) [18].

On the other hand, the challenging wound treatment situation might be improved by introducing composite dressings with inner layers designed to support epidermis regeneration [19,20]. Furthermore, multilayer polyelectrolyte shells may be applied as the constructs for scaffolds for supporting immobilized cells’ adhesion and functioning for cell and/or tissue repair or replacement [21]. Immobilization of cells within multilayered shells can concentrate biological material in proper space, enable its functioning for biological processes regulation purposes.

The polyelectrolyte shells for scaffolding systems can be applied in cooperation with different cells (e.g., neuronal cells) for bandages applications [22]. For example, the alginate/poly(L-lysine) bilayer scaffolds were reported to allow for the survival of rat neurons in culture for over 2 weeks what can find application in controllable regeneration of neural networks [9]. The photo cross-linked hyaluronic acid (HA) hydrogels modified with poly(L-lysine) (PLL) and HA multilayer films were reported to serve for NIH-3T3 fibroblast attachment [23]. Meng and co-workers proposed chitosan/alginate/hyaluronic acid polyelectrolyte composite sponges, cross-linked with genipin. The authors were able to confirm the effectiveness of their composite material in wound healing in animal studies [24].

Among different membrane scaffolds materials, polymeric hydrogels stand for their good biocompatibility and structural similarity to tissue. However, they are characterized by weak mechanical strength and a lack of biological activity. Recently, the incorporation of metallic nanoparticles into biomaterials has been explored to increase mechanical strength while adding additional antibacterial and/or antiviral features [25,26]. Composite materials have gained significant attention in wound healing in the last years. Dressings based on polyelectrolyte multilayers follow this trend.

This study aims to develop multifunctional polyelectrolyte layers enhanced by hydroxyapatite (HAP), gold nanoparticles (AuNPs), and/or fullerenol (FUOL) nanocomposites to achieve a wound dressing applied on soft and hard tissue borders. We designed, prepared, and characterized the novel bilayers for supporting wound healing and granulation tissue formation, simultaneously protecting the defect against significant fibrosis. In addition, the designed bilayers might be used as an innermost layer of composed dressings.

Poly(ethyleneimine) (PEI) as one of the construction elements was selected due to its features as a carrier, broadly used in drug and gene delivery systems used in regenerative medicine, especially wound healing [27,28,29,30]. Hydroxyapatite is broadly known for facilitating wound healing by stimulating collagen production [31] and usage as a scaffolding material [32]. Another inorganic substance—fullerenol—was applied to provide additional interactions with its OH group and strengthen the shell structure. We incorporated gold nanoparticles in the system structure to ensure the antimicrobial properties of the system.

To assess the usability of dressings, the constructed bilayers were examined using microscopic techniques like transmission electron (TEM), scanning electron (SEM), and fluorescence microscopies. Moreover, we perform the high-resolution transmission electron microscope (HRTEM) in STEM (scanning transmission electron microscope) mode, coupled with an energy dispersive X-ray detector (EDX) to confirm gold nanoparticles’ presence. Then, the cytocompatibility of the designed layers on HDF and hFOB cell lines was evaluated. Moreover, the morphology of the systems of cells immobilized within designed shells was assessed.

## 2. Materials and Methods

*Reagents*: poly(ethyleneimine), branched, Mn ~60,000, Mw 750,000, analytical standard, 50% (*w*/*v*) in H_2_O (Sigma-Aldrich, Munchen, Germany); gold nanoparticles, 100 ppm, of 10 nm size, stabilized with sodium citrate, in 0,01% Tween 20 (UTTC UW: Bell Synthesis, Warsaw, Poland); hydroxyapatite, aqueous paste, particle size <50 nm, 30 wt. %, spec. surface area ≥80 m^2^/g (Sigma-Aldrich); polyhydroxy small gap fullerenes, hydrated (Sigma Aldrich, UE); propidium iodide (Sigma-Aldrich), trypsin EDTA solution C (0.5%), EDTA 0.2% (10×) (Biological Industries, Kibutz Beit HaEmek, Israel) phosphate-buffered saline (PBS) (Biomed Lublin, Lublin, Poland), MilliQ water. 

*Media*: Fibroblast Growth Medium (Sigma-Aldrich); Ham’s F12 Medium/Dulbecco’s Modified Eagle’s Medium (F12/DMEM) (Gibco, Thermo Fisher Scientific, Waltham, MA, USA); RPMI1640 (Euroclone, Pero, Italy), Fetal Bovine Serum (FBS) (Sigma).

*Culture medium*: RPMI1640 supplemented with 10% FBS, 1% antibiotics (Gibco, Thermo Fisher Scientific, Waltham, MA, USA). 

*Cells*: Human dermal fibroblasts (HDF) cell line (Sigma-Aldrich); human fetal osteoblasts (hFOB 1.19) cell line (CRL-11372™, ATCC^®^, Manassas, VA, USA).

### 2.1. Preparation of Multilayered Films

In this study, multilayer films containing gold nanoparticles and/or fullerenol were designed and prepared based on polyethylenimine (PEI). Briefly, the membranes were obtained using a layer-by-layer method described in our previous works [33,34] (See Supplementary Materials Schema S1). First, the solution of PEI was made at a concentration of 1 mg/mL in PBS. Then, the single, as well as bilayered membranes, were prepared.

To obtain the composite of polyethylenimine with AuNPs (PEI-Au), 20 ppm AuNPs solution in PBS was added to PEI at a 1:1 ratio and subsequently stirred for four hours at room temperature. To obtain the polyethylenimine with AuNPs composite and fullerenol complex (PEI-Au-FUOL), the fullerenol solution in PBS at concentration 0.5 mg/mL was added at a 1:1 ratio to the PEI-Au solution prepared of 40 ppm AuNPs solution in PBS and PEI at a 1:1 ratio and subsequently stirred for four hours at room temperature.

The 0.6% hydroxyapatite solution in PBS was added to fullerenol solution in PBS at a concentration of 0.5 mg/mL at a 1:1 ratio and subsequently stirred for four hours at room temperature to get the hydroxyapatite with fullerenol membrane complex (HAP-FUOL).

Next, the bilayered membranes were made by the deposition of the second film on the basic ones. For this purpose: (1) the HAP layer of 0.3% hydroxyapatite solution in PBS was deposited on PEI-Au layer to obtain the polyethylenimine with AuNPs|hydroxyapatite membrane (PEI-Au|HAP), (2) the HAP layer of 0.3% hydroxyapatite solution in PBS was deposited on PEI-Au-FUOL layer—to obtain the polyethylenimine incorporating AuNPs and fullerenol|hydroxyapatite membrane (PEI-Au-FUOL|HAP), (3) the HAP-FUOL layer was deposited on PEI-Au layer—to obtain the polyethylenimine with AuNPs| hydroxyapatite with fullerenol membrane (PEI-Au|HAP-FUOL).

The AuNPs conjugated with fluorescein were applied for membrane forming to assess the internalization of NPs by cells.

All membrane layers were deposited on the support—sterile coverslips. For that purpose, the slips were placed in a polyelectrolyte solution for 30 min. Then, dried, washed twice, and transferred to the culture wells.

### 2.2. Characterization of Multilayered Shells and Systems of Cells Immobilized within the Shells

#### 2.2.1. Wettability of Membrane Shell

We analyzed the wettability of polyelectrolyte membranes adsorbed on the glass support using a Phoenix 150 surface energy analyzer (Surface Electro-Optics Haas, Suwon, Korea) and dedicated IMAGE XP software (SEO Software, Suwon, Korea). The measurements were performed at room temperature.

#### 2.2.2. Transmission Electron Microscopy

Transmission electron microscopy (TEM) analysis of the designed membranes was performed on aTalos F200X transmission microscope (FEI, Waltham, MA, USA) at 200 kV. We applied TEM and STEM mode using high-angle annular dark-field imaging to conduct measurements. Energy-dispersive X-ray spectroscopy (EDX) was performed on a BD4 instrument (Bruker, Billerica, MA, USA) to detect the metallic nanoparticles. Moreover, the EDX-spectra was used for the compositional analysis of given samples. All TEM samples were deposited on a copper grid coated with a carbon holey film.

### 2.3. Culture of HDF and hFOB Cells

HDF cells were grown under standard culture conditions (5% CO_2_, 37 °C) in fibroblast growth medium. In contrast, the hFOB 1.19-line cells were cultured in F12/DMEM culture medium supplemented with 10% fetal calf serum and geneticin. Cells were grown to approximately 80% confluency. The medium was then removed from the culture bottles, the cells were washed with PBS without calcium and magnesium ions, and trypsinized with trypsin with EDTA.

The HDF cells were placed on tested membranes at a density of 3 × 103 cells per well (1.6 × 10^3^/cm^2^). The hFOB 1.19 cells were placed on tested membranes at a density of 5 × 10^3^ cells per well (2.6 × 10^3^/cm^2^). As a negative control, the cells were cultured in the presence of support without deposited membranes. Cell viability was assessed after 3, 6, and 10 days of culture in a cytochemical reaction with propidium iodide in a flow cytometer.

### 2.4. Fluorescence Staining

For fluorescence staining, cells immobilized within membranes deposited on glass coverslips after 3, 6, and 10 days of culture were fixed in 4% paraformaldehyde (PFA) in PBS at room temperature (20 °C) for 15 min. Then, cell membranes were permeabilized using TRITON X100 detergent to allow dyes to penetrate individual cells. The next step was the addition of fluorochrome-conjugated phalloidin staining F-actin. Phalloidin is a toxin isolated from a phylum Amanita fungus (*Amanita phalloides*), which binds directly to filamentous actin (F-actin) found in large quantities in fibroblasts. Then DAPI solution—fluorochrome specifically staining DNA—was added to the cells to visualize single cells. Under UV light, cell nuclei stained DAPI shows blue fluorescence. After three washes in PBS, cells were photographed using an IX70 fluorescence microscope (Olympus, Tokyo, Japan). Blue DAPI fluorescence (λ = 460 ÷ 500 nm) and red phalloidin fluorescence (λ = 570 nm) images were examined.

### 2.5. Flow Cytometric Analysis

The eukaryotic cell presence was assessed using a Canto II flow cytometer (Becton Dickinson Immunocytochemistry Systems, Franklin Lake, NY, USA). The results were processed by the FACS Diva software system (Becton Dickinson, Franklin Lake, NY, USA). Evaluated objects were separated from other events based on the light scattering characteristics.

### 2.6. Scanning Electron Microscopy Analysis

The cells immobilized within membranes were visualized using a scanning electron microscope (SEM); (TM 1000, Hitachi, Tokyo, Japan). After 3, 6, and 10 days of culture, cells were fixed with 2.5% glutaraldehyde. Then, the fixed samples were rinsed several times with Milli Q water and placed for 15 min in 75.0% ethanol. The procedure was repeated. The next stage was a 15-min incubation of samples in 99.8% ethanol. Then, the samples were air-dried and placed on microscope measuring chamber.

### 2.7. Statistical Analysis

All data are expressed as mean ± standard deviation (SD). The mean values and standard deviations and the significance of differences were calculated in the Statistica 7.1 software (TIBCO Software, Palo Alto, CA, USA). Values where *p* < 0.05 were assumed to be significant.

## 3. Results and Discussion

### 3.1. Characterization of Multilayered Shells and Systems of Cells Immobilized within the Shells

#### Wettability Assessment of Membrane Shells

**Figure 1:**
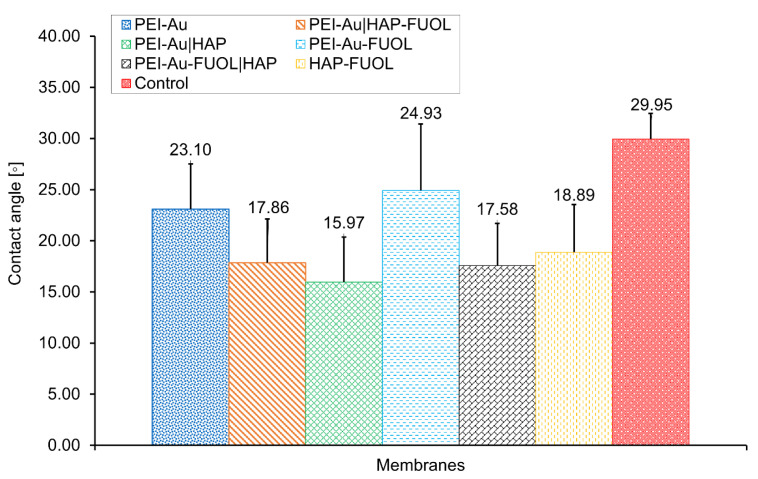
The water contact angle of the composite membranes. Key to the symbols: PEI-Au—polyethylenimine incorporating gold nanoparticles; PEI-Au-FUOL—polyethylenimine incorporating gold nanoparticles and fullerenol, hydroxyapatite mixed with FUOL; bilayered: PEI-Au|HAP—polyethylenimine incorporating gold nanoparticles and hydroxyapatite; PEI-Au|HAP-FUOL—polyethylenimine incorporating gold nanoparticles, and hydroxyapatite incorporating FUOL; PEI-Au-FUOL|HAP—polyethylenimine incorporating gold nanoparticles and FUOL layer and hydroxyapatite layer. The values are presented as mean ± SD.

Analysis of the material in terms of wettability (hydrophilicity or hydrophobicity) allows, among others, for its assessment towards suitability for biomedical applications. Basing on the analysis of the water contact angle of the membranes: polyethylenimine incorporating gold nanoparticles (PEI-Au), polyethylenimine incorporating gold nanoparticles and fullerenol (PEI-Au-FUOL), and bilayer membranes PEI-Au|HAP, PEI-Au-FUOL|HAP, PEI-Au|HAP-FUOL with a second layer built of HAP or HAP-FUOL, it can be stated that there were no significant differences in the values obtained for them. The surfaces of all evaluated membranes exhibited a water contact angle below 90°, indicating the hydrophilic character (Figure 1).

### 3.2. Study of the Structure of Developed Membrane Shells

#### Surface Characterization of the Designed Membrane Shells

**Figure 2:**
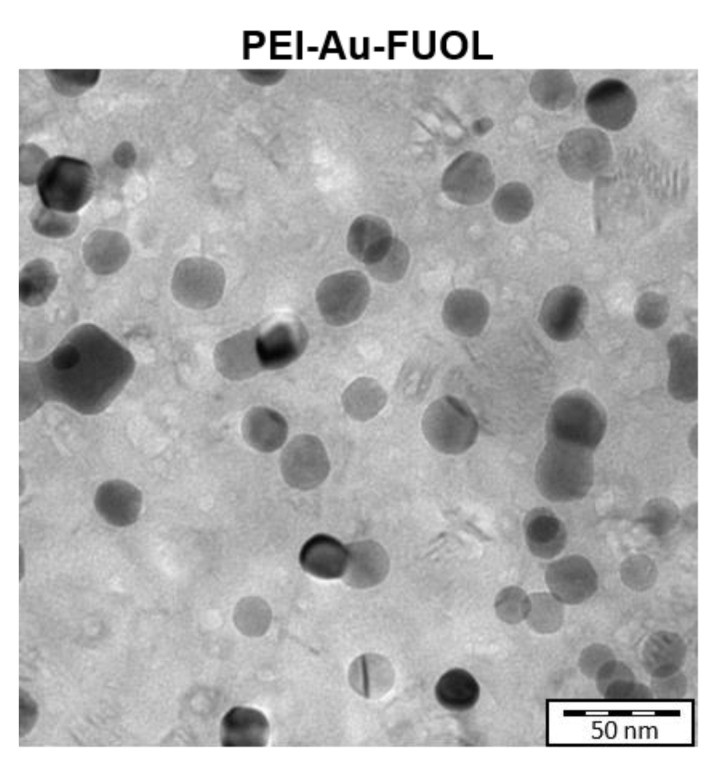
The surface of polyethylenimine incorporating gold nanoparticles and fullerenol (PEI-Au-FUOL) in transmission electron microscopy.

Transmission electron microscopy (TEM) may be used for observing the developed wound dressing in a one-layer form to confirm the presence of gold nanoparticles in the membrane structure. The TEM visualization of exemplary PEI-Au-FUOL surface is shown in Figure 2.

Dark gray, circular structures are AuNPs embedded in fullerenol and polyethyleneimine matrix (light gray). Several cavities in film can be seen as whitish (very light gray) spots. One can see that gold nanostructures are of relatively uniform size with good agreement to the size declared by the producer. Moreover, AuNPs are also evenly distributed within layers with no tendency to aggregate.

**Figure 3:**
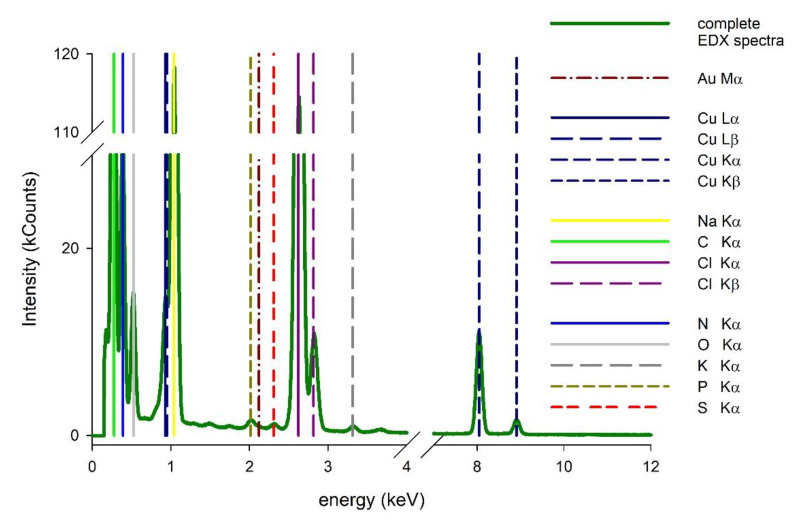
EDX spectra of PEI-Au-HAP membrane with Au peaks visible.

In membranes with AuNPs involving the HAP layer, the NPs presence could not be confirmed as the nanoparticles’ view was overlapped with HAP. For that reason, we used a high-resolution transmission electron microscope (HRTEM) in STEM (scanning transmission electron microscope) mode. The applied microscope was coupled with an energy dispersive X-ray mapping (EDX) instrument, which allowed us to confirm the presence of gold nanoparticles and for mapping of them. As a result, only the slight peak corresponding to Au was observed in EDX spectra of the PEI-Au-HAP membrane (Figure 3).

**Figure 4:**
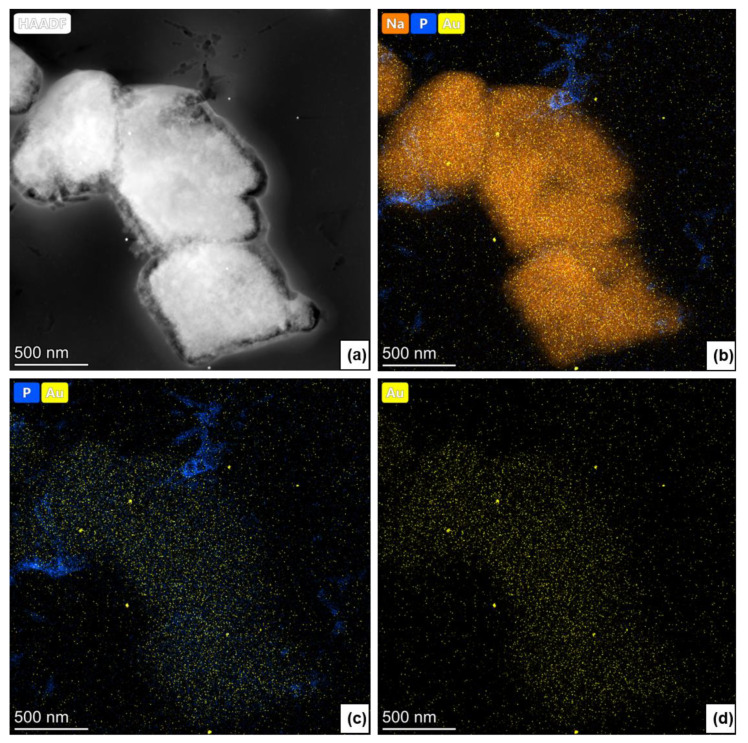
(**a**) STEM image of the HAP|PEI-Au-FUOL; (**b**) corresponding EDX map of the Na, P, Au distribution; (**c**) corresponding EDX map of the P, Au distribution; (**d**) corresponding EDX map of the Au distribution.

Furthermore, using a scanning transmission electron microscope (STEM), we were able to visualize the distribution of AuNPs within the membrane. Figure 4a shows the EDX spectrum of PEI-Au-FUOL|HAP membrane: the gold nanoparticles are visible as bright spots, proving their presence and explaining why the signal observed in EDX spectra was of low intensity.

Furthermore, the presence of the other elements like Na (being a part of the stabilizer of Au), P (as a substrate of HAP), Cu, and C (not presented here) was observed on the studied surface. (Figure 4b–d).

### 3.3. Cytotoxicity of the Membrane Shells

The developed wound dressing was tested for in vitro cytotoxicity on HDF human dermal fibroblasts and hFOB human fetal osteoblastic cell lines. Cells immobilized within the membranes deposited on glass slides were cultured for 10 days. As a negative control, served cells cultured in the presence of slides without membranes during 10 days of culture. Cell density and morphology were evaluated both quantitatively by flow cytometry and qualitatively by brightfield and fluorescent microscopy.

After 10-days of hFOB cells culture, no significant difference in the percentage of viable cells was observed between control and cells cultured within PEI-Au, PEI-Au|HAP, PEI-Au-FUOL, PEI-Au-FUOL|HAP. On the contrary, there was a significant difference between control and membranes PEI-Au|HAP-FUOL and HAP-FUOL. Nonetheless, the difference in percentage share did not exceed 14%. There were meanly 85% cells alive.

**Figure 5:**
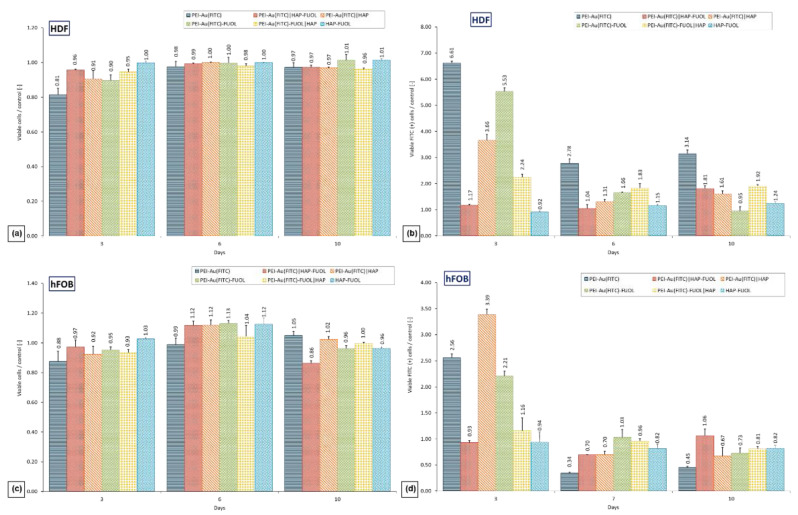
Percentage rate to the control of viable (**a**) HDF or (**b**) HDF FITC (+) and (**c**) hFOB hFOB or (**d**) hFOB FITC (+) cells maintained in the presence of polyelectrolyte membranes after 1-, 5- and 10-day culture. The values are presented as the mean ± SD. Abbreviations: Au, gold nanoparticles; FITC, Fluorescein isothiocyanate; FUOL, fullerenol; HAP, hydroxyapatite; HDF, Human Dermal Fibroblasts; PEI, polyethylenimine.

After 10 days of immobilized HDF cell culture, the mean viable cell percentage was equal to 91%. In comparison, the viable cell (average) means share was 93% in the control (Figure 5a,c).

We noticed certain regularities during the examination of the fluorescence of HDF cells maintained in the presence of the designed membranes. After 3 days of the experiment, the fluorescence was statistically higher in cells immobilized within all membranes involving Au NPs conjugated with fluorescein than in the control group and cells immobilized within the HAP-FUOL membranes. It can be noted that control HDF cells exhibited fluorescence. After three days, the percentage share of cells FITC (+) declines during culture (on 6-th and 10-th day) for all membranes involving AuNPs conjugated with fluorescein except for bilayer membranes involving FUOL, where the share increased on the 6-th day.

When examining the fluorescence of hFOB cells immobilized within tested membranes, we observed that after three days of culture, the cells immobilized within all tested membranes involving AuNPs conjugated with fluorescein except bilayer membranes involving FUOL exhibited statistically higher fluorescence compared to the control group. There was no statistical difference between the immobilized within PEI-Au|HAP-FUOL or PEI-Au-FUOL|HAP cells FITC (+) and the control. After 6- and 10-day cultures, there was no statistical difference in the percentage share of FITC (+) cells compared with control (Figure 5b,d). It was shown that the internalization of Au NPs did not cause a cytotoxic effect on evaluated cells.

#### 3.3.1. Scanning Electron Microscopy Analysis

**Figure 6:**
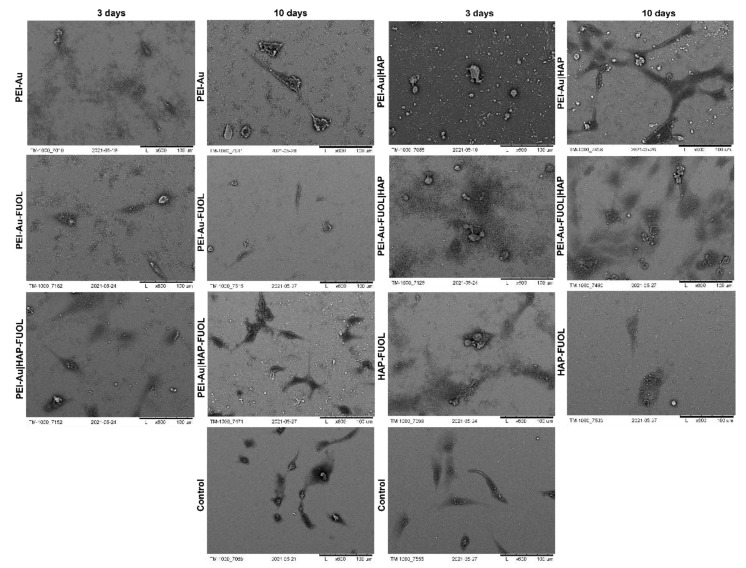
SEM visualization of hFOB cells immobilized within designed scaffolds after 3- or 10-day culture. As a control, served cells cultured on glass slides without the membranes. Key to the symbols: PEI-Au: the membrane built of the polyethylenimine with AuNPs incorporating; PEI-Au|HAP: bilayer consisting of polyethylenimine with AuNPs incorporating and hydroxyapatite; PEI-Au-FUOL: polyethylenimine with AgNPs and FUOL incorporating; PEI-Au-FUOL|HAP bilayer consisting of polyethylenimine with AuNPs and FUOL incorporating and hydroxyapatite; PEI-Au|HAP-FUOL bilayer consisting of polyethylenimine with AuNPs incorporating and hydroxyapatite with FUOL incorporating; HAP-FUOL: hydroxyapatite with FUOL incorporating. Scale bar: 100 µm.

**Figure 7:**
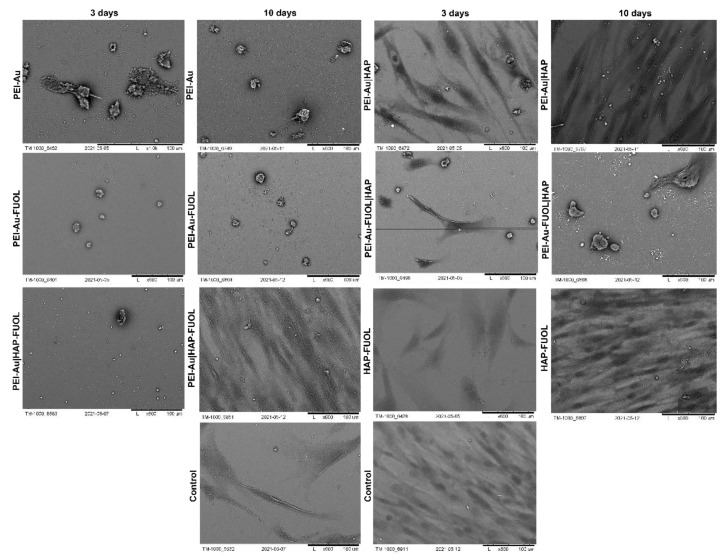
SEM visualization of HDF cells immobilized within designed scaffolds after 3- or 10-day culture. As a control, served cells cultured on glass slides without the membranes. Key to the symbols: PEI-Au: the membrane built of the polyethylenimine with AuNPs incorporating; PEI-Au|HAP: bilayer consisting of polyethylenimine with AuNPs incorporating and hydroxyapatite; PEI-Au-FUOL: polyethylenimine with AgNPs and FUOL incorporating; PEI-Au-FUOL|HAP bilayer consisting of polyethylenimine with AuNPs and FUOL incorporating and hydroxyapatite; PEI-Au|HAP-FUOL bilayer consisting of polyethylenimine with AuNPs incorporating and hydroxyapatite with FUOL incorporating; HAP-FUOL: hydroxyapatite with FUOL incorporating. Scale bar: 100 µm.

The morphology of cells immobilized within the developed membranes was examined using scanning electron microscopy (SEM). Wherein the cells grown directly on a slide without membranes served as control. The images of the systems after 3- and 10-days of culture are shown in Figure 6, Figure 7, Figures S1 and S2.

After 3 days of culture, the HDF cells immobilized within PEI-Au or PEI-Au-FUOL exhibited a spherical shape. On the other hand, the cells immobilized within PEI-Au|HAP shown spindle shape; however, in that case, a small number of spherical shape cells was also observed. The PEI-Au-FUOL|HAP membrane promotes the formation of both spindle and spherical shape cells. The spherical cells were observed on PEI-Au|HAP-FUOL membranes. On the HAP-FUOL membranes, the spindle shape cells with some number of spherical ones were visible.

After 10 days of culture, the cells maintain within PEI-Au or PEI-Au-FUOL remained in their spherical shape. On the contrary, the cells immobilized within PEI-Au|HAP exhibited a spindle shape. However, many spherical shape cells were observed similarly to the surfaces examined after 3 days. PEI-Au-FUOL|HAP membrane still promoted the formation of both spindle and spherical shape cells. Although the spherical cells were observed on PEI-Au|HAP-FUOL membranes on the third day, the cells’ morphology was comparable with the control after 10 days of culture.

After 3 days of culture, the hFOB cells immobilized within PEI-Au exhibited a spherical shape. In addition, the cells immobilized within PEI-Au-FUOL formed both spindle and spherical shapes. The cells immobilized within PEI-Au|HAP showed a spherical shape. Whereas PEI-Au-FUOL|HAP membranes promote spindle shape cells with a small number of spherical ones. The spherical and spindle cells were also noticed on PEI-Au|HAP-FUOL membranes. On the HAP-FUOL membranes, the spindle shape cells, and spherical ones were observed.

After 10 days of culture, the hFOB cells immobilized within PEI-Au still exhibited a spherical shape. The cells immobilized within PEI-Au-FUOL formed both spindle and spherical shape like in three days culture. Conversely, the cells immobilized within PEI-Au|HAP changed their previous spherical shape to spindle one. PEI-Au-FUOL|HAP still promotes spindle shape cells with a small number of spherical cells. The spherical and spindle cells were also observed on PEI-Au|HAP-FUOL membranes. On the HAP-FUOL membranes, the spindle shape cells, and spherical ones were still present.

The introduction of AuNPs into the layer causes both hFOB and HDF cells to form a spherical shape instead of a spindle, characteristic of fibroblastoid cells. On the other hand, in the case of hFOB cells, FUOL addition to the layer involving AuNPs (PEI-Au) induces spindle-shaped cells besides spherical ones.

**Figure 8:**
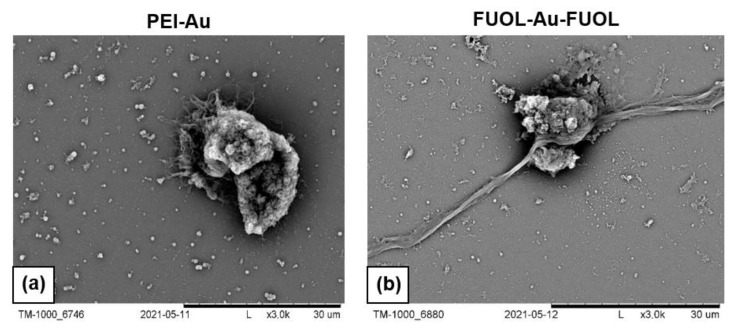
SEM visualization of HDF cells immobilized within designed scaffolds after 10-day culture. *Key to the symbols*: (**a**) PEI-Au: polyethylenimine with AuNPs incorporating; (**b**) PEI-Au-FUOL: polyethylenimine with AgNPs and FUOL incorporating. Magnification: 3000×.

In immobilized HDF cell cultures, although the cells became spherical after 10 days of maintenance for PEI-Au and PEI-Au-FUOL membranes, cells grown on PEI-Au-FUOL showed protrusions that might facilitate confluence (Figure 8).

The involvement of the HAP layer induces the appearance of spindle-shaped HDF and hFOB cells besides spherical ones. Conversely, incorporating FUOL into the HAP layer slows the spindle cell appearance in HDF cells.

**Figure 9:**
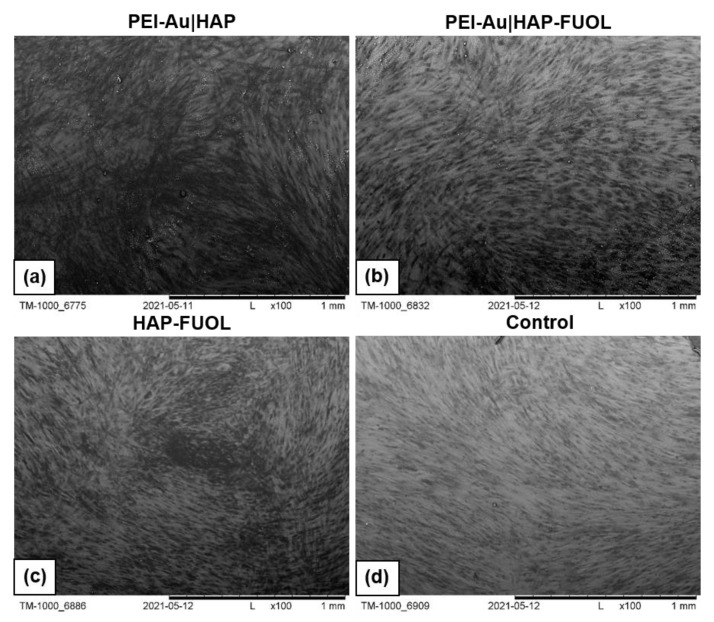
SEM visualization of HDF cells immobilized within designed scaffolds after 10-day culture. *Key to the symbols*: (**a**) PEI-Au|HAP: bilayer consisting of polyethylenimine with AuNPs incorporating and hydroxyapatite; (**b**) PEI-Au|HAP-FUOL bilayer consisting of polyethylenimine with AuNPs incorporating and hydroxyapatite with FUOL incorporating; (**c**) HAP-FUOL: hydroxyapatite with FUOL incorporating; (**d**) Control: cells cultured on glass slides without the membranes. Magnification ×100.

After 10 days of culture of HDF cells immobilized within designed scaffolds, i.e., HAP-FUOL, PEI-Au|HAP, PEI-Au|HAP-FUOL, similar growth patterns to the control group were observed (Figure 9). Moreover, after 10 days of culture, the hFOB cells immobilized within the PEI-Au|HAP-FUOL scaffold showed similar growth patterns to the control group (Figure 7).

#### 3.3.2. Fluorescence Microscopy Analysis

The morphology of hFOB cells immobilized within the exemplary shells blocking or not AuNPs internalization was examined using fluorescent microscopy. Cell nuclei are visualized by staining with DAPI blue fluorescent dye; the cell cytoskeleton is stained red with F-Actin.

**Figure 10:**
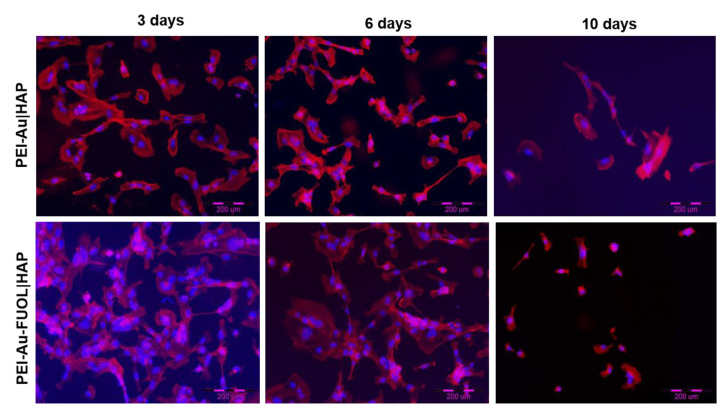
Visualization of cultured hFOB cells grown on a PEI-Au|HAP and PEI-Au-FUOL|HAP membranes after 3, 6, and 10 days. The nuclei of cells are stained in blue. The red fluorescence shows F-Actin. *Abbreviations*: Au, gold nanoparticles; HAP, hydroxyapatite, hFOB, Human Fetal Osteoblasts; PEI, polyethylenimine.

The microscopic fluorescence images of hFOB cells immobilized within the PEI-Au|HAP membrane unblocking the AuNPs internalization or PEI-Au-FUOL|HAP membrane blocking internalization during 10 days of culture are presented in Figure 10.

No differences in morphology of hFOB cells immobilized within two types of membranes were observed during the culture. Moreover, the morphology of hFOB cells visualized using fluorescence microscopy confirmed the morphology visualized using SEM.

## 4. Discussion

The designed membrane scaffolds incorporating bacteriostatic elements and elements supporting blockage their internalization were examined as materials for dressings usage. It has been shown previously [35] that introducing AuNPs into the layer causes the immobilized fibroblastic cells to form a spherical shape instead of a spindle, characteristic of fibroblastoid cells. At the present experiment, that property was observed in both cell lines hFOB and HDF.

FUOL addition to the PEI-Au layer incorporating AuNPs promotes spindle-shaped cells besides spherical ones in hFOB cell culture. At the same time, the involvement of the HAP layer induces the appearance of spindle-shaped cells besides spherical ones in both HDF and hFOB cultures. The spindle form taken by the cells would indicate that the membranes involving HAP AuNPs facilitating cells to form the shape characteristic for the adhesion phase and allowing for higher adhesion of immobilized cells comparing with the HAP-free membranes. Nevertheless, the spherical nature of the cells does not disqualify the scaffold for cell maintenance, while the protrusions allow for confluence. It can be noted that the cells in their native tissue show different morphologies than those grown on a support. The applied membrane scaffolds seem to affect cellular morphology. The mechanism by which a cell can translate its shape into an intracellular signal needs a local molecular measurement. To unambiguously define this relationship, it would be necessary to know the transduction mechanism that integrates local signals from the cell’s shape into its physiology [36].

The PEI-Au|HAP-FUOL or PEI-Au-FUOL|HAP membranes were observed to block the internalization of FITC elements of membranes by immobilized eukaryotic hFOB cells. On the other side, AuNPs’ internalization did not affect the viability of the cells. Moreover, the membranes ensured the counts and morphology of hFOB or HDF cells compared with control.

## 5. Conclusions

The designed bilayer membranes with incorporated FUOL, involving bacteriostatic elements, preventing internalization of bacteriostatic elements by hFOB cells, simultaneously ensuring the proper counts and morphology of maintained within eukaryotic cells can be recommended for usage as a multilayer composed within dressings at the bone-skin interface.

## Data Availability

Not applicable.

## Supplementary Materials

The following are available online at https://www.mdpi.com/article/10.3390/membranes11080565/s1.
