# Supplementary material for: A Composite Membrane System with Gold Nanoparticles, Hydroxyapatite, and Fullerenol for Dual Interaction for Biomedical Purposes
Source: Membranes (Basel). 2021 Jul 27;11(8):565. doi: 10.3390/membranes11080565 (PMC8398639; doi:10.3390/membranes11080565)
Supplement: Supplementary file 1 [file membranes-11-00565-s001.zip › membranes-1294062-supplementary.pdf]

Supplementary Materials: A Composite Membrane System with Gold Nanoparticles, Hydroxyapatite, and Fullerenol for Dual Interaction for Biomedical Purposes

Anna Grzeczkowicz [1], Monika Drabik [1], Agata Lipko [1], Paweł Bącal [1,2], Angelika Kwiatkowska [1], Beata Kazimierczak [1] and Ludomira H. Granicka [1,*]

[1] Nalecz Institute of Biocybernetics and Biomedical Engineering, Polish Academy of Sciences, Trojdena 4 st., 02-109 Warsaw, Poland; agrzeczkowicz@ibib.waw.pl (A.G.); mdrabik@ibib.waw.pl (M.D.);
alipko@ibib.waw.pl (A.L.); pbacal@ibib.waw.pl (P.B.); ankwiatkowska@op.pl (A.K.); bkazimierczak@ibib.waw.pl (B.K.)
[2] Institute of Paleobiology, Polish Academy of Sciences, Twarda 51/55 st, 02-109 Warsaw, Poland
* Correspondence: lgranicka@ibib.waw.pl; Tel.: +48-22-592-59-00

Preparation of Multilayered Films

Procedure: Conjugation of gold nanoparticles to FITC

1. Fluorescein isothiocyanate (FITC) was dissolved in acetone at a concentration of 1 mg/mL.
2. The FITC solution was added to the suspention of gold nanoparticles (160 ppm) by volume
1. 10 μL per 1 mL of gold nanoparticle suspension.
2. Tubes with a mixture of gold nanoparticles and FITC were left overnight at 4 °C.
3. To confirm the FITC presence, the absorbance of the samples (Au-FITC) was measured at a wavelength of λ = 450 nm using a Multiskan FC photometer (Thermo Fisher Scientific).

Schema: The illustration presenting the composition of studied membranes.

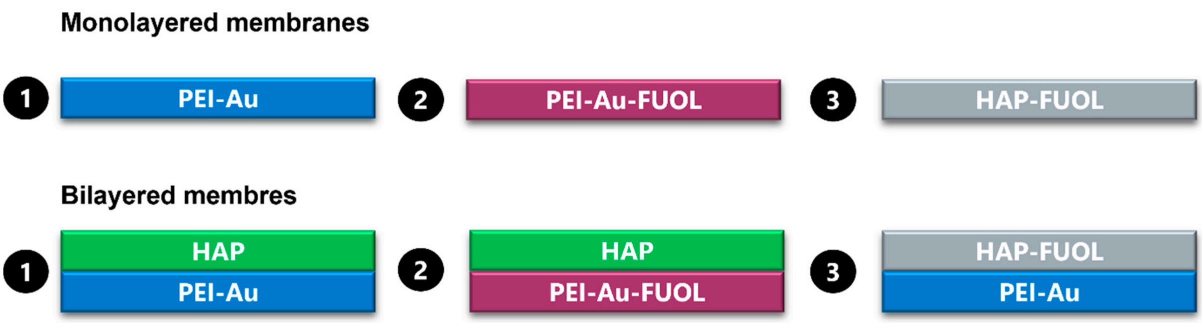

Schema S1. The illustration showing the composition of studied films.

Scanning Electron Microscopy Analysis

The morphology of cells immobilized within the developed membranes was examined using scanning electron microscopy (SEM). Wherein the cells grown directly on a slide without membranes served as control. The images of the systems after 3- and 10-days of culture are shown in Figures S1, S2.

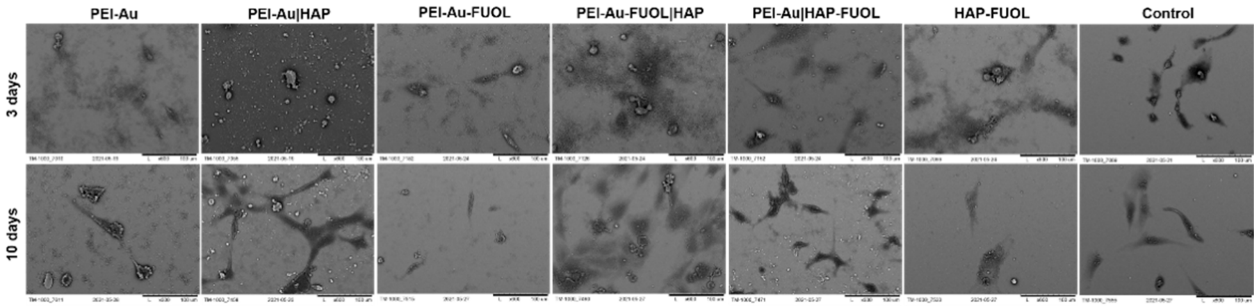

Figure S1. SEM visualization of hFOB cells immobilized within designed scaffolds after 3- or 10-day culture. As a control, served cells cultured on glass slides without the membranes. Key to the symbols: PEI-Au: the membrane build of the polyethylenimine with AuNPs incorporating; PEI-Au|HAP: bilayer consisting of polyethylenimine with AuNPs incorporating and hydroxyapatite; PEI-Au-FUOL: polyethylenimine with AgNPs and FUOL incorporating; PEI-Au-FUOL|HAP bilayer consisting of polyethylenimine with AuNPs and FUOL incorporating and hydroxyapatite; PEI-Au|HAP-FUOL bilayer consisting of polyethylenimine with AuNPs incorporating and hydroxyapatite with FUOL incorporating; HAP-FUOL: hydroxyapatite with FUOL incorporating.

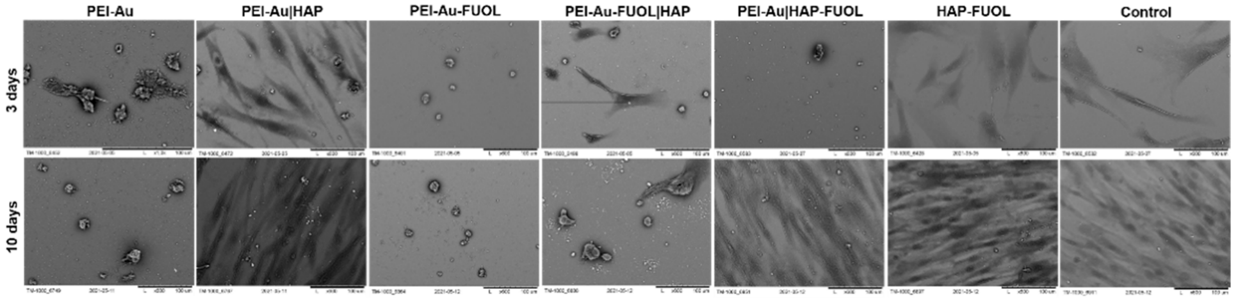

Figure S2. SEM visualization of HDF cells immobilized within designed scaffolds after 3- or 10-day culture. As a control, served cells cultured on glass slides without the membranes. Key to the symbols: PEI-Au: the membrane build of the polyethylenimine with AuNPs incorporating; PEI-Au|HAP: bilayer consisting of polyethylenimine with AuNPs incorporating and hydroxyapatite; PEI-Au-FUOL: polyethylenimine with AgNPs and FUOL incorporating; PEI-Au-FUOL|HAP bilayer consisting of polyethylenimine with AuNPs and FUOL incorporating and hydroxyapatite; PEI-Au|HAP-FUOL bilayer consisting of polyethylenimine with AuNPs incorporating and hydroxyapatite with FUOL incorporating; HAP-FUOL: hydroxyapatite with FUOL incorporating.
